# Vitamin D levels and risk of juvenile idiopathic arthritis: A Mendelian randomization study

**DOI:** 10.1002/acr.24815

**Published:** 2022-11-26

**Authors:** Sarah LN Clarke, Ruth E Mitchell, Gemma C Sharp, Athimalaipet V Ramanan, Caroline L Relton

**Affiliations:** 1 MRC Integrative Epidemiology Unit, University of Bristol, Bristol UK; 2 School of Population Health Sciences, Bristol Medical School, University of Bristol, Bristol, UK; 3 Department of Paediatric Rheumatology, Bristol Royal Hospital for Children, Bristol, UK; 4 School of Translational Health Sciences, Bristol Medical School, University of Bristol, Bristol, UK

**Keywords:** Juvenile idiopathic arthritis, Vitamin D, 25-(OH)D, Mendelian Randomization

## Abstract

**Objectives:**

Observational studies report mixed findings regarding the association between vitamin D and JIA incidence or activity, however such studies are susceptible to considerable bias. Since low vitamin D levels are common within the general population and easily corrected, there is potential public health benefit in identifying a causal association between vitamin D insufficiency and JIA incidence. To limit bias due to confounding and reverse causation we examined the causal effect of the major circulating form of vitamin D, 25-(OH)D, on JIA incidence using Mendelian randomization (MR).

**Methods:**

In this two sample MR analysis we used summary level data from the largest and most recent genome wide association study (GWAS) of 25-(OH)D levels (sample size 443,734), alongside summary data from two JIA GWASs (sample sizes 15,872 and 12,501), all from European populations. To test and account for potential bias due to pleiotropy we employed multiple MR methods and sensitivity analyses.

**Results:**

We found no evidence of a causal relationship between genetically predicted 25-(OH)D levels and JIA incidence (OR 1.00, 95% CI 0.76-1.33 per standard deviation increase in standardised natural-log transformed 25-(OH)D levels). This estimate was consistent across all methods tested. Additonally there was no evidence that genetically predicted JIA causally influences 25-(OH)D levels (0.004 standard deviation decrease in standardised natural-log transformed 25-(OH)D levels per doubling odds in genetically predicted JIA, 95% CI -0.009-0.002).

**Conclusion:**

Given the lack of a causal relationship between 25-(OH)D levels and JIA, population level vitamin D supplementation is unlikely to reduce JIA incidence.

## Introduction

JIA is the most common rheumatic disease affecting children, with a prevalence of approximately 1/1000^1^. JIA is considered a complex autoimmune disease, influenced by both environmental and genetic factors. Whilst there is a growing appreciation of genetic influences^2–4^ on JIA, we have much less clarity regarding environmental effects on disease risk.^5^ Identification of modifiable environmental factors which predispose to JIA or can be used to risk stratify or alter disease outcome has huge potential benefit for patients.

**Figure 1:**
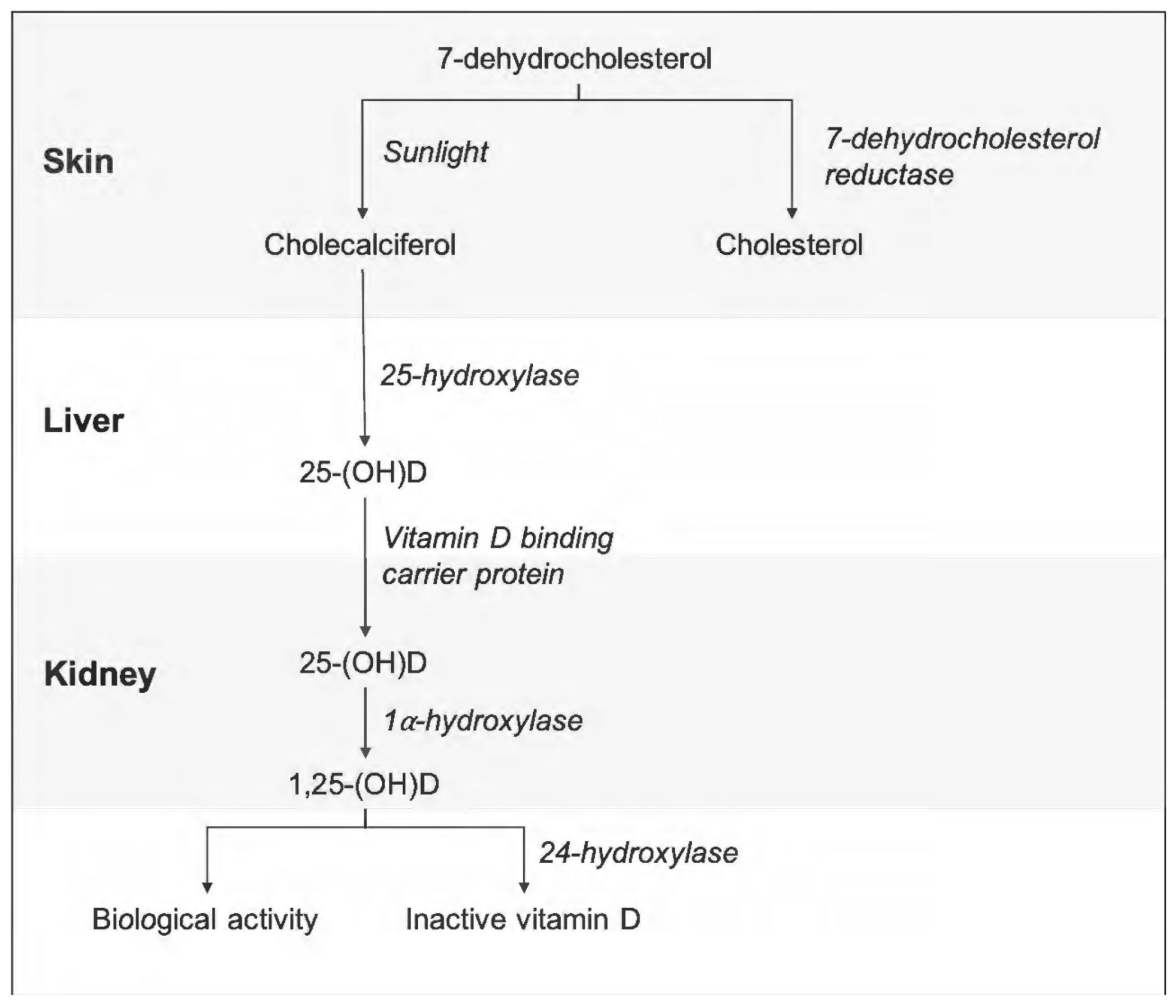
Major steps in the vitamin D synthetic pathway. 7-dehydrocholesterol in the skin is converted to cholecalciferol (pre-vitamin D) in the presence of sunlight. Cholecalciferol is then hydroxylated in the liver to 25-(OH)D (calcidiol). 25-(OH)D is bound by vitamin D binding protein and transported to the kidneys where it undergoes 1-hydroxylation to its active form - 1,25-(OH)D (calcitriol). Downstream effects of biologically active vitamin D are mediated via the vitamin D receptor. Vitamin D is catabolised through a variety of oxidation steps involving 24-hydroxylase.

Observationally, low vitamin D levels have been associated with increased incidence and disease activity of many other autoimmune disorders.^6,7^ Vitamin D is a fat-soluble steroid hormone whose synthesis requires multiple steps involving biologically inactive precursors (Figure 1). The major circulating form of vitamin D measured in blood is 25-(OH)D, however the downstream derivative 1,25-(OH)_2_D is the biologically active form. Vitamin D levels are predominantly influenced by sunlight; ultraviolet light induces conversion of 7-dehydrocholesterol to pre-vitamin D3 in the skin. Some vitamin D is also obtained via the gut from diet and exogenous supplementation. Vitamin D acts via the vitamin D receptor (VDR) and plays a key role in calcium homeostasis and bone metabolism. However the VDR is also extensively expressed on immune cells and vitamin D has been shown to have a number of immune-modulatory effects.^7,8^

The role of vitamin D in JIA has recently been examined in a scoping review.^9^ The studies included in this review predominantly examine the role of vitamin D in a) JIA activity/disease course or b) report vitamin D status of JIA patients versus healthy controls after disease onset, with mixed results. Further literature review identified few published studies examining the vitamin D status of JIA patients prior to disease onset. Thorsen *et al*^10^ found no evidence of association between cord blood 25-(OH)D levels and later JIA risk (adjusted OR 1.2 95% CI 0.9-1.6) in 300 matched case-control pairs, however observational studies of vitamin D levels and JIA risk at other pre-disease timepoints are lacking. Conversely, a recent study of 202 JIA case-control pairs found a potentially protective association between maternal prenatal or early childhood ultraviolet radiation (UVR) exposure and JIA, with vitamin D proposed as a mediator.^11^ Thus the role of vitamin D in JIA incidence remains unclear.

Vitamin D insufficiency (25-(OH)D <50nmol/L) is increasingly common within the general population, with children being at particular risk in part due to the nutritional demands associated with periods of rapid growth.^12^ Therefore establishing a causal relationship between vitamin D and JIA has important implications. Determining causal relationships from observational data is challenging; associations may be confounded (e.g. by medication use such as steroids), subject to reverse causation (e.g. patients with JIA may have altered behaviour and spend less time outdoors due to their disease state), or subject to recall bias. Mendelian randomization (MR) is a method of causal inference which relies on genetic data to interrogate the causal relationship between an exposure and outcome of interest, where single nucleotide polymorphisms (SNPs) are used as genetic proxies for the exposure of interest. The reliance of MR on genetic variants, assigned at the point of conception and not influenced by lifetime environmental exposures and experiences, makes these analyses robust to reverse causation and confounding compared to traditional epidemiological studies. The value of this approach can be seen in relation to multiple sclerosis (MS) where observation associations between MS and vitamin D (examined as latitudinal gradient^13^, vitamin D levels^14^ and VDR polymorphisms^15^) have been replicated in MR studies of MS and vitamin D.^16–18^

The aim of this bidirectional, two sample Mendelian randomization study is to examine the causal relationship between vitamin D and JIA using the latest genome wide association study (GWAS) summary data.

## Methods

### Data sources and selection of genetic instruments

**Figure 2:**
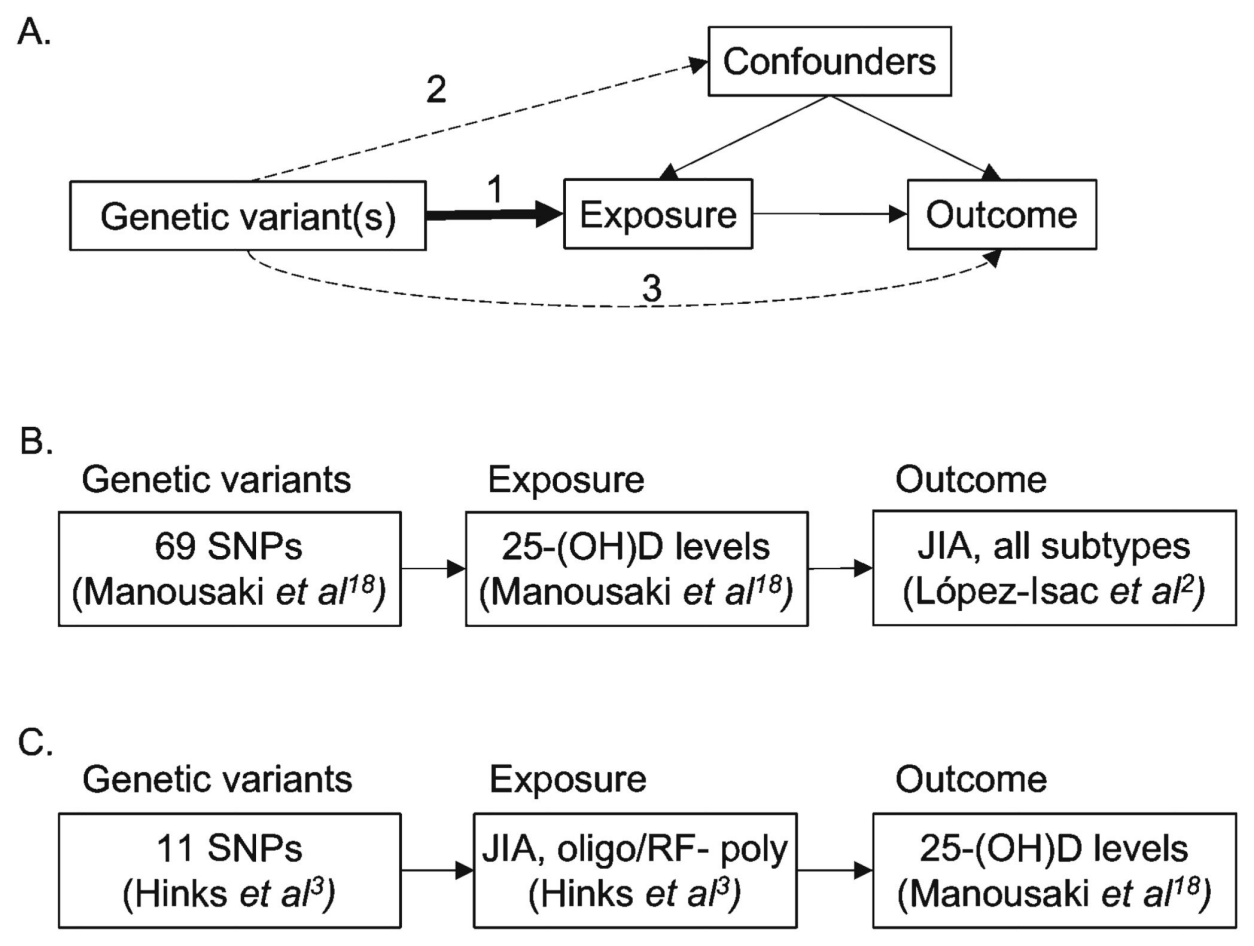
Mendelian randomization (MR) analyses undertaken in this study. A) diagram of the assumptions for a Mendelian randomization (MR) analysis. In order to be valid MR instruments genetic variants must 1) be strongly associated with the exposure of interest (relevance assumption), 2) cannot be associated with confounders of the exposure or the outcome of interest (independence assumption) and 3) cannot influence the outcome except via the exposure (exclusion restriction). B) Forward MR analysis examining the causal association between genetically predicted vitamin D levels and JIA. C) Reverse MR analysis examining the association between JIA and vitamin D levels. Data sources are shown in parentheses. Oligo, oligoarticular; RF-poly, rheumatoid factor negative polyarticular.

The first assumption of MR is that genetic variants are strongly associated with the exposure of interest (Figure 2). Therefore the genetic variants selected for vitamin D comprised the 69 lead, common, conditionally independent SNPs that were robustly associated with 25-(OH)D levels (P value <6.6x10^-9^) from a large GWAS meta-analysis of 25-(OH)D in Europeans (N=443,734, 52.7% female) adjusted for age, sex, genotype array, genotype batch, vitamin D supplementation and latitude.^19^ The more stringent P value threshold of 6.6x10^-9^, rather than the traditional 5x10^-8^, was derived from an earlier bespoke analysis of the vitamin D GWAS cohort^20^ and thus was retained for this analysis. Publicly available summary data from the most recent JIA GWAS study was used as the JIA outcome dataset in this analysis due to it’s broad genomic coverage and inclusivity of JIA subtypes.^2^ This dataset comprises 3,305 JIA patients (all subtypes) and 9,196 healthy controls of European ancestry.

To investigate the causal effect of JIA genetic liability on vitamin D levels we derived a genetic instrument for JIA as an exposure from an Immunochip study comprising 2,816 oligoarticular and RF^-^ polyarticular JIA cases (76.6% female) and 13,056 healthy controls (55.1% female) of European ancestry.^3^ This dataset comprises the largest sample size of any publicly available JIA dataset and identifies the greatest number of replicated JIA-associated loci. The instrument comprised 11 conditionally independent SNPs (r^2^ <0.001) strongly associated with JIA (P value <5x10^-8^) using an additive genetic model (Supplementary Table 1). Full summary data from the vitamin D GWAS study^19^ was made available by the authors to allow for bidirectional MR analysis with vitamin D as an outcome.

Details of the genetic variants used for each instrument can be found in Supplementary Table 1.

### Statistical analyses

Two sample Mendelian randomization (2SMR), implemented in the *TwoSampleMR* package^21^ in the R software platform (version 4.0.2)^22^, was used to examine the causal relationship between genetically predicated vitamin D levels and JIA. Effect estimates for the selected vitamin D SNPs were extracted from the JIA outcome dataset. Where a SNP was not available in the outcome dataset, suitable LD proxies (r^2^ >0.8) were identified using the *LDLinkR* package^23^ from LDLink^24^ (Supplementary Table 2). Alleles were harmonised between exposure and outcome datasets to ensure SNP-exposure and SNP-outcome effects correspond to the same allele. We aligned palindromic SNPs using default thresholds; non-inferable SNPs were excluded to limit effect allele ambiguity between datasets. We used the inverse variance weighted method (IVW) to estimate the causal effect of 25-(OH)D on JIA susceptibility (odds ratio per one standard deviation (SD) increase in standardised natural-log transformed 25-(OH)D risk). Briefly, the IVW method pools the SNP-exposure and SNP-outcome associations in a random effects meta-analysis weighted by the inverse variance of the SNP-outcome association. For clinical context, we have expressed one SD increase in natural log transformed 25-(OH)D levels as the corresponding change in nmol/L at clinically relevant 25-(OH)D levels − vitamin D deficient (<25nmol/L), insufficient (25-50nmol/L) and sufficient (50-70nmol/L, Table 1). The analysis was also performed in reverse, i.e. with JIA as the exposure and vitamin D levels as the outcome, using the JIA genetic instrument and the vitamin D GWAS summary dataset, to estimate the causal effect of JIA genetic risk on vitamin D levels. As JIA is a binary exposure, the MR estimate refers to the SD change in standardised natural-log transformed 25-(OH)D levels per logOR increase in genetically predicted JIA. To aid interpretability, these MR estimates were multiplied by 0.693 to represent SD change in standardised natural-log transformed 25-(OH)D levels per doubling in odds of genetic liability to JIA.^25^ Instrument strength was measured using the F-statistic whereby the higher the F-statistic the lower the risk of weak instrument bias. This was calculated as $F=\left(\frac{N-K-1}{K}\right)\left(\frac{R^{2}}{1-R^{2}}\right)$, where *N* indicates sample size, *K* indicates number of SNPs in the instrument and *R^2^* indicates the proportion of the variance in the exposure explained by the instrument.

### Sensitivity analyses

The second MR assumption requires that genetic instruments are not associated with confounders which could bias the association between the exposure and the outcome (Figure 2). To assess this, we queried both the vitamin D and the JIA genetic instruments in the *PhenoScanner* database^26^. We defined strong associations as those traits associated with a SNP below a P value threshold Bonferroni-corrected for the number of SNPs in the genetic instrument i.e. P <7.25x10^-4^ (0.05/69) for vitamin D and P <4.54x10^-3^ (0.05/11) for JIA. Traits identified below the defined P value thresholds in *Phenoscanner* (“secondary traits”) were grouped into 21 trait categories. None of the SNP-trait associations identified with *PhenoScanner* were felt to be potential confounders of the association between vitamin D and JIA in our analyses, and therefore violate the second MR assumption.

The third MR assumption states that genetic variants must be associated with the outcome only via the exposure (exclusion-restriction), i.e. that there are no horizontally pleiotropic effects. The inclusion of multiple SNPs within the genetic instrument increases the statistical power of the analysis. However, this also increases the risk of including more pleiotropic genetic variants and violating the exclusion-restriction criteria. We undertook sensitivity analyses to attempt to detect and account for potential pleiotropic effects. Heterogeneity can indicate pleiotropy which would violate the core assumptions of MR, thus heterogeneity was assessed using Cochran’s Q statistic.^27^ To further investigate potential horizontal pleiotropy we undertook MR-Egger regression^28^, weighted median estimation^29^ and Radial MR.^30^ MR-Egger regression can provide an accurate estimate of causality even if all IVs are invalid. MR-Egger analysis uses the inverse variance of SNP-outcome associations as weights in a weighted linear regression of SNP-outcomes effects on SNP-exposure effects, however unlike the IVW method, the intercept is not constrained to zero. The slope of this regression represents the casual effect estimate and, in the absence of pleiotropy, equals the IVW estimate. The intercept can be interpreted as the average pleiotropic effects of the IVs, with directional pleiotropy being indicated by an intercept other than zero. Regression dilution I^2^ (GX)^31^ was assessed to ensure the MR-Egger analyses was robust, with a value of >90% considered low risk of bias. The weighted median estimator offers protection against invalid IVs/horizontal pleiotropy by providing a consistent estimate of causality when up to 50% of the weight comes from invalid IVs. It estimates the weighted median value of the Wald ratio. Radial MR is used to identify potentially outlying SNPs within the genetic instrument and provide MR estimates using alternative weights. We note the caution from the authors of the vitamin D meta-analysis about potential pleiotropic effects when using the 69 vitamin D associated SNPs in a MR context. To further examine whether pleiotropic SNPs may be biasing the association between vitamin D and JIA we undertook a sensitivity analysis excluding all non-specific genetic instruments (SNPs which associated with any secondary trait identified in PhenoScanner, Supplementary Table 1 and 3). Additionally we undertook a second sensitivity analysis using only the seven SNPs from six loci previously robustly associated with vitamin D, five of which are in close proximity to genes and which are directly involved in the vitamin D biological pathway (Figure 1, Supplementary Table 1). Consistent estimates across all MR methods (IVW, MR-Egger, weighted median and Radial MR) and vitamin D instruments make bias due to pleiotropy less probable.

## Results

### Two sample MR analysis examining the causal effect of vitamin D on JIA risk

**Figure 3:**
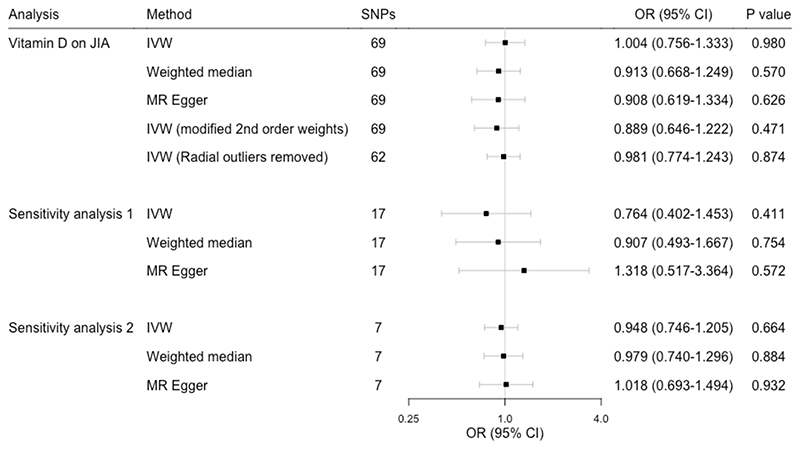
Two sample Mendelian Randomization analysis showing the effects of genetically predicted 25-(OH)D levels on JIA. Sensitivity analysis 1 is restricted to SNPs not associated with a secondary trait using PhenoScanner and sensitivity analysis 2 is restricted to SNPs previously associated and predominantly with the vitamin D synthetic pathway. Odds ratios represent the odds of JIA per one standard deviation increase in natural-log transformed 25-(OH)D. CI, confidence intervals; IVW, inverse variance weighted; OR, odds ratio; SNPs, single nucleotide polymorphisms.

Sixty-nine vitamin D associated SNPs were used as instruments with sufficient strength for MR analysis (F-statistic 115.7, R^2^ 0.018). There was no evidence that higher genetically predicted 25-(OH)D levels are causally associated with reduced JIA risk (OR 1.00, 95% CI 0.76-1.33 per SD increase in standardised natural-log transformed 25-(OH)D levels, Figure 3). We also conducted a “leave-one-out” analysis which found that no single SNP was driving the MR estimates (Supplementary Figure 1). Based on the JIA sample size of 12,501, alpha of 0.05, case fraction of 0.264 and R^2^ of 0.018, this study has 80% power to detect effects as small as an OR of 1.47 per standard deviation change in standardised natural-log transformed 25-(OH)D levels, and 100% to detect OR 1.80 per standard deviation change in standardised natural-log transformed 25-(OH)D levels.

### Assessment of heterogeneity and control for pleiotropy

We found no strong evidence of pleiotropy when examining the MR-Egger intercept (intercept 0.005, P value 0.45, Supplementary Table 5) and no evidence of asymmetry in the funnel plot of single SNP effects (Supplementary Figure 2). However, Cochran’s Q statistic indicated evidence of heterogeneity (Q statistic 99.33, P value 0.008), Supplementary Table 6) which can suggest pleiotropy, therefore we undertook additional analyses to assess and account for potential pleiotropic effects. MR-Egger regression and the weighted median estimator provided MR estimates comparable to the IVW estimate (Figure 3). Radial MR was used to identify and adjust for outlying and thus potentially pleiotropic SNPs; the IVW estimate was recalculated using a) first orders weights with removal of seven outlying SNPs identified using radial regression and b) modified second order weights with all 69 SNPs (Supplementary Tables 4 and 7). In both cases, MR estimates were comparable to the original IVW estimate (Figure 3). To further validate the effect of genetically predicted vitamin D on JIA, sensitivity analyses were undertaken using subsets of the genetic instrument for vitamin D levels. Firstly, we used a 17 SNP vitamin D instrument (Supplementary Table 1) where all SNPs strongly associated with any secondary trait within *PhenoScanner* (P value <7.24 x 10^-4^) were excluded (i.e. high stringency removal of non-specific genetic instruments, sensitivity analysis 1). Secondly, we used an instrument of seven 25-(OH) D genetic variants robustly associated with vitamin D levels from earlier GWAS studies (Supplementary Table 1) which are also predominantly involved in the vitamin D biological pathway (sensitivity analysis 2). Again comparable MR estimates to the main IVW estimate were obtained (Figure 3).

### Reverse two sample MR analysis examining the causal effect of JIA on vitamin D levels

**Figure 4:**
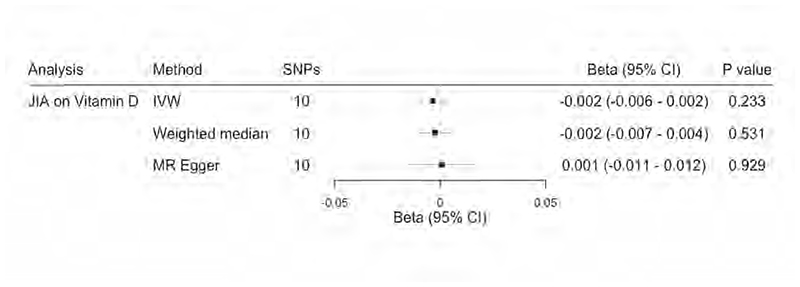
Two sample Mendelian Randomization analysis showing the effects of genetically predicted JIA on 25-(OH)D levels. Beta coefficients represent standard deviation change in standardised natural-log transformed 25-(OH)D levels per doubling in odds of genetic liability to JIA. CI, confidence intervals; IVW, inverse variance weighted; SNPs, single nucleotide polymorphisms.

To assess whether the absence of a causal relationship between vitamin D levels and JIA in our previous analysis despite some observational evidence to the contrary could be due to reverse causation (i.e. JIA causes changes in vitamin D levels), we used a genetic instrument comprising 11 SNPs associated with oligoarticular and RF- polyarticular JIA (Supplementary Table 1). This instrument has sufficient strength for MR analysis (F-statistic 52.6, R^2^ 0.035). It comprises SNPs which were not associated with a confounding secondary trait in *PhenoScanner* (Supplementary Table 8). There was no evidence that genetically predicted oligoarticular and RF- polyarticular JIA is associated with changes in 25-(OH)D levels (0.004 SD decrease change in standardised natural-log transformed 25-(OH)D levels per doubling odds in genetically predicted JIA, 95% CI -0.009-0.002, Figure 4). Cochran’s Q statistic found no evidence of heterogeneity (Q statistic 9.35, P value 0.75, Supplementary Table 5) and the MR-Egger intercept indicated low risk of bias due to pleiotropy (intercept -0.001, P value 0.60, Supplementary Table 5. MR estimates were comparable across the IVW, weighted median estimator and MR-Egger methods. Leave-one-out analysis showed that no single SNP was biasing the MR estimates (Supplementary Figure 3).

## Discussion

Using summary level data from the most recent 25-(OH)D^19^ and JIA^2,3^ GWAS studies in European populations, we undertook the first two sample MR study in JIA to investigate the presence and magnitude of the causal effect of vitamin D on disease risk. Despite utilising multiple, complementary MR methodologies and sensitivity analyses, we found no evidence of a causal relationship between genetically predicted vitamin D levels and JIA (all subtypes). We were also unable to demonstrate a causal effect of genetically predicted oligoarticular and RF- polyarticular JIA on circulating vitamin D levels.

A causal role for low vitamin D in increasing multiple sclerosis risk has been robustly established using MR methodology^16–18^, however the results of our study are in keeping with the growing number of other MR studies which have found no causal relationship between vitamin D and other immune-mediated disorders such as RA^32^, SLE^32^, inflammatory bowel disease^33^, type 1 diabetes^34^, childhood asthma^35,36^ and eczema^36,37^. Therefore, although we cannot exclude small effects of vitamin D levels on JIA incidence, our results show that higher genetically predicted vitamin D levels do not reduce JIA risk and that attempts to increase circulating vitamin D levels, e.g. through supplementation, are unlikely to substantially reduce the incidence of JIA in European populations.

The role of vitamin D in JIA outcome has also been considered within the literature with regards to the level of disease activity^38,39^ and incidence of JIA-associated complications e.g. uveitis^38^. However, corresponding trial data of vitamin D supplementation is lacking with regard to reducing disease activity^40^. Accordingly, our study has not demonstrated a causal effect of genetically predicted oligoarticular and RF- polyarticular JIA on circulating vitamin D levels. However, exploring the precise role of vitamin D in JIA disease activity in a two sample MR context would require datasets which identify genetic variants proxying JIA outcome within a JIA population. Such datasets are not currently available, thus this study cannot exclude a role for vitamin D in JIA severity, disease activity or prognosis and thus vitamin D supplementation may have a role as a treatment adjunct in JIA.

The major strength in this study lies within the rigorous interrogation of the association between vitamin D and JIA across multiple analyses, utilising the most up to date and comprehensive datasets available for vitamin D and JIA. We used stringent criteria for inclusion of SNPs as instrumental variables in our analyses (MR assumption 1) and also undertook comprehensive assessment for confounding (MR assumption 2). Furthermore, we employed multiple pleiotropy robust MR methods and vitamin D instruments to attempt to identify and account for any biasing effects of horizontal pleiotropy on our findings (MR assumption 3). Nevertheless, this study has its limitations. Firstly, the datasets we used derive from different age populations − the vitamin D dataset derives from an adult population and the JIA datasets from paediatric ones − therefore this study is assuming that the genetic effects on the exposure of interest are fixed and constant across the life-course. Whilst large scale paediatric vitamin D GWAS data is not available to formally test this assumption, the replication of some of the adult GWAS vitamin D associations in a small study of children from the general population at age 6 years and age 14 years provides reassurance that vitamin D genetic risk is constant across the ages.^41^ The generalisability of this data to non-European populations is also limited given the sparsity of ethnically diverse GWAS summary data, therefore extrapolation of these findings to populations of non-European ancestry should be cautious. Whilst we have attempted to identify and account for bias due to pleiotropy, we cannot exclude residual bias where the function of the included SNPs largely remains unknown. The sample size of the current JIA outcome dataset (total sample size =12,501), whilst considerable for a rare disorder, does limit the power of this analysis, thus it is possible that vitamin D has a causal effect on JIA that is too small to be detectable by our study. However, the clinical relevance and utility of identifying such a small causal effect, given the overall population prevalence of JIA, is questionable. Additionally, the JIA outcome dataset is heterogenous, including all JIA subtypes to maximise sample size and power. This makes it possible that any causal effect of vitamin D on JIA risk which is either subtype specific and/or has opposing effects by subtype would be masked within this study; subtype specific JIA datasets suitable for MR analysis are not currently available. Finally, since this study examines the effect of the major circulating form of vitamin D, 25-(OH)D, on JIA we are unable to interrogate a causal relationship between other components of the vitamin D pathway, particularly biologically active vitamin D, and JIA.

## Conclusions

In conclusion, this study represents the first MR study to investigate a potential causal effect of vitamin D on JIA disease risk. This study demonstrates no evidence of a causal relationship between genetically predicted 25-(OH)D levels and JIA. Similarly, there was no evidence that genetically predicted oligoarticular and RF-polyarticular JIA is causally associated with altered 25-(OH)D levels. Accordingly, this study suggests that low vitamin D levels do not increase JIA risk and that vitamin D supplementation would not reduce the incidence of JIA in the general population. Further interrogation of these findings with larger, ethnically diverse and subtype specific datasets would be beneficial. In addition, the role of vitamin D in JIA disease activity and thus the utility of vitamin D supplementation as a treatment adjunct, warrants further investigation.

## List of List of abbreviations

25-(OH)D: 25-hydroxy vitamin D
CI: confidence interval
GWAS: genome wide association study
IVW: inverse variance weighted
JIA: juvenile idiopathic arthritis
MR: Mendelian randomization
MS: multiple sclerosis
OR: odds ratio
SD: standard deviation
SNPs: single nucleotide polymorphisms
UVR: ultraviolet radiation
VDR: vitamin D receptor

## Supplementary Material

Supplementary Figues 1-3

Supplementary Tables 1-9

## Figures and Tables

**Table 1:** Effect of a one SD increase in standardised natural-log transformed 25-(OH)D levels at clinically relevant vitamin D thresholds, expressed on the nmol/L scale. Of note, for vitamin D insufficient individuals (25-50nmol/L), a one SD increase in standardised natural log 25-(OH)D would result in normalisation of the vitamin D level (>50nmol/L).

Clinically relevant vitamin D threshold	Effect of one SD change in standardised natural log transformed 25-(OH)D at given threshold in nmol/L
Vitamin D deficient (<25nmol/L)	14.61nmol/L
Vitamin D insufficient (25-50nmol/L)	29.22nmol/L
Vitamin D sufficient (50-70nmol/L)	40.91nmol/L

## Data Availability

The datasets analysed during the current study are available in the GWAS Catalog repository (Studies GCST90010715, GCST010144 and GCST005528); www.ebi.ac.uk/gwas/studies/GCST90010715, www.ebi.ac.uk/gwas/studies/GCST010144 and www.ebi.ac.uk/gwas/studies/GCST005528.^2,3,19^

## Significance and Innovation

- Observational data regarding the association between vitamin D levels and JIA is mixed, and is susceptible to confounding and reverse causation
- Mendelian randomization is a method of causal inference which is less susceptible to these biases and was used to examine the causal relationship between vitamin D and JIA risk
- We found no evidence of a causal relationship between genetically predicted vitamin D levels and JIA, suggesting observational data supporting this association could be confounded
- Our findings suggest that population level vitamin D supplementation will not reduce JIA incidence
