# Supplementary material for: Vitamin D levels and risk of juvenile idiopathic arthritis: A Mendelian randomization study
Source: Arthritis Care Res (Hoboken). Author manuscript; Available in PMC 2024 Dec 9. (PMC7617134; doi:10.1002/acr.24815)

Supplementary figure legends

Supplementary figure 1: Leave one out analysis. To test whether any single SNP is driving the association between vitamin D and JIA, a leave one out analysis was conducted. Each row represents 2SMR analysis of vitamin D on JIA using all 69 SNPs in the 25-(OH)D instrument except the SNP listed on the y-axis. The point represents the MR effect size (beta coefficient) and the bars represent 95% confidence intervals. The row in red represents the IVW estimate when all SNPs are included in the analysis.

Supplementary figure 2: Leave one out analysis. To test whether any single SNP is driving the association between JIA and vitamin D, a leave on out analysis was conducted. Each row represents 2SMR analysis of JIA on vitamin D using all SNPs in the JIA instrument except the SNP listed on the y-axis. The point represents the MR effect size (beta) and the bars represent 95% confidence intervals. The row in red represents the IVW estimate when all SNPs are included in the analysis.

Supplementary figure 3: Funnel plot of 25-(OH)D single SNP MR effect estimates (beta) on the x-axis against inverse standard error (y-axis) for the IVW, MR-Egger and weighted median estimator analyses.

Supplementary Figure 1

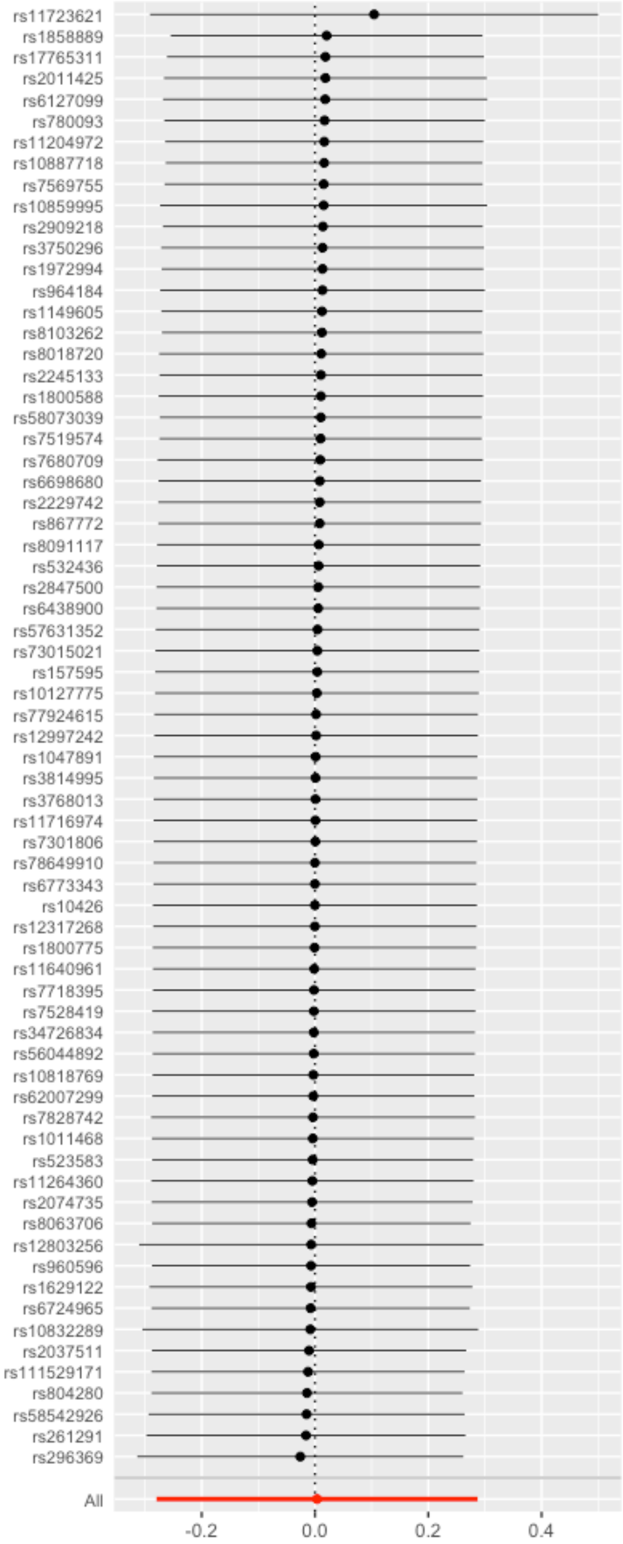

Supplementary Figure 2

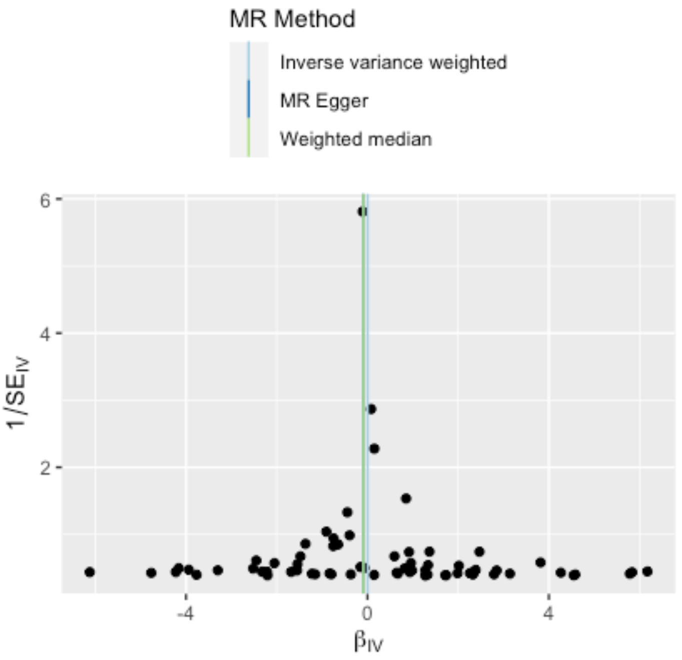

Supplementary Figure 3

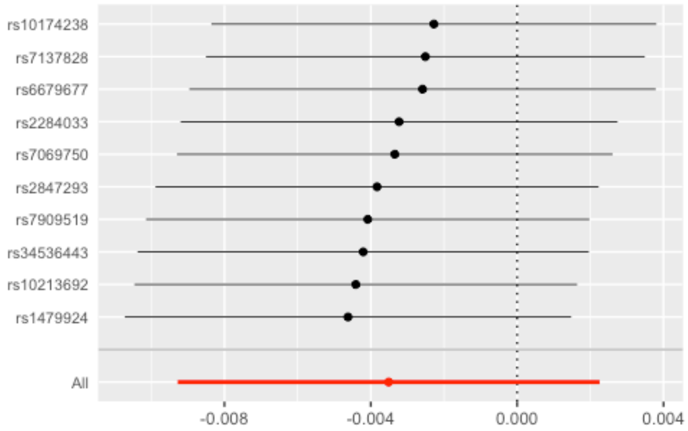

Supplement: Supplementary Figues 1-3 [file EMS194713-supplement-Supplementary_Figues_1_3.pdf]
